# Lateral Route Endoscopic Thyroidectomy with gas Insufflation: Proposed Critical View of Safety

**DOI:** 10.1007/s00268-023-06977-8

**Published:** 2023-03-27

**Authors:** Islam A. Elzahaby, Mohamed Hamdy, Ahmed Abdallah

**Affiliations:** Assistant Professor of Surgical Oncology, Mansoura Oncology Center (OCMU), Faculty of Medicine, Mansoura University, 60 Gomhoria Street, Mansoura, 35511 Egypt

## Abstract

**Background:**

The extra-cervical lateral route endoscopic thyroidectomy (LRET) approaches such as the trans-axillary, breast and axillo-breast approaches are proved to be safe, feasible, esthetic, highly effective. The inherent difficulty and long learning curve of these techniques prevents its widespread application.

**Methods:**

Benefiting from the experience of more than 5 years in LRET approaches with CO_2_ insufflation, the authors developed ten surgical key steps and a critical view of safety (CVS) for performing thyroid lobectomy via LRET approaches. A detailed description and a video of the surgical technique is provided.

**Results:**

Application of these structured key steps and CVS was feasible and effective in achieving thyroid lobectomy in all selected cases with unilateral goiter up to 8 cm, even in cases with thyroiditis or controlled toxic adenoma, without any adverse events and with shorter operative time than the non-structured surgical technique.

**Conclusion:**

The described ten key steps and CVS are conclusive, applicable, easy to learn. Our video could act as a guide for promoting the standardized, safe, and wide application of LRET techniques.

**Supplementary Information:**

The online version contains supplementary material available at 10.1007/s00268-023-06977-8.

## Introduction

Lateral route endoscopic thyroidectomy (LRET) approaches, particularly the axillary and the axillo-breast approaches are performed with high degree of success in several institutions worldwide. [1,2] The long learning curve of the various ET techniques underscores the need for structured teaching and standardization of the procedure.

Based on the literature review, our own clinical experience (of more than 190 cases of LRET) and our previous publications in the field of endoscopic thyroidectomy, parathyroidectomy, submandibular sialadenectomy and lastly cervical lymph node dissection, we introduce in detail the surgical key steps and a proposed CVS for performing LRET with gas insufflation to promote its safety and its standardized application in clinical settings. The procedure, called Elzahaby’s ten key steps together with the critical butterfly view of safety, is described here:

## Surgical technique

The procedure, called Elzahaby’s ten key steps together with the critical butterfly view of safety, is described here:

*Step 1:* Patient position and drawings

Under general anesthesia, the patient is placed in Kocher’s position with the ipsilateral arm supported in abduction position.

**Fig. 1:**
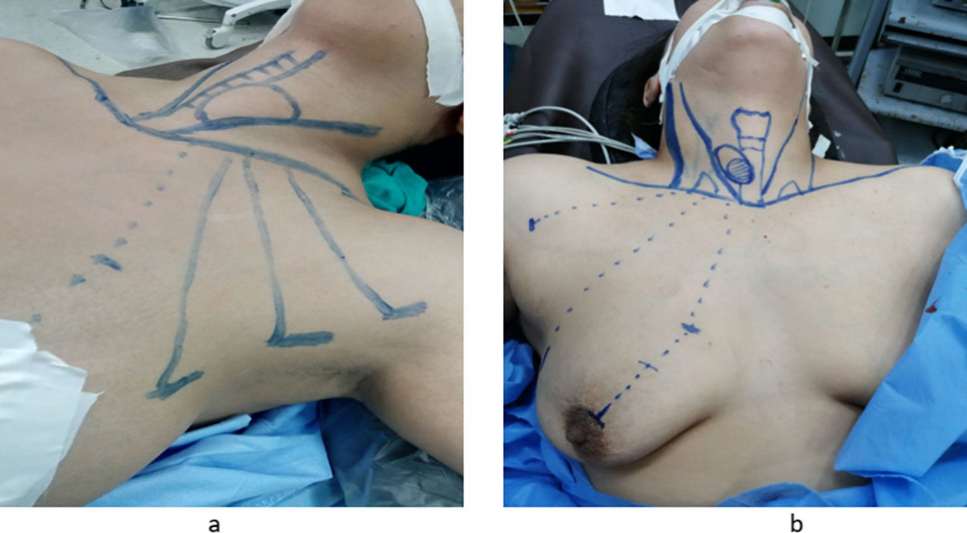
**a** Preoperative markings of the port sites in the trans-axillary ET approach. **b** Preoperative markings of the port sites in the unilateral axillo-breast ET approach

Preoperative markings of the port sites vary according to the utilized approach as follows: in axillary approach, the three ports are placed below the anterior axillary line equidistant apart with the 12 mm camera port in the middle (Fig. 1a), whereas in unilateral axillo-breast approach, two ports are placed below the anterior axillary line (the lower one is for the12 mm camera port) and a third port in the upper edge of the ipsilateral areola. In patients with large, ptotic or lactating breasts, this third 5 mm port could be shift up outside breast mound in the parasternal area. (Fig. 1b).

*Step 2:* Port placement and creation of the surgical working space

**Fig. 2:**
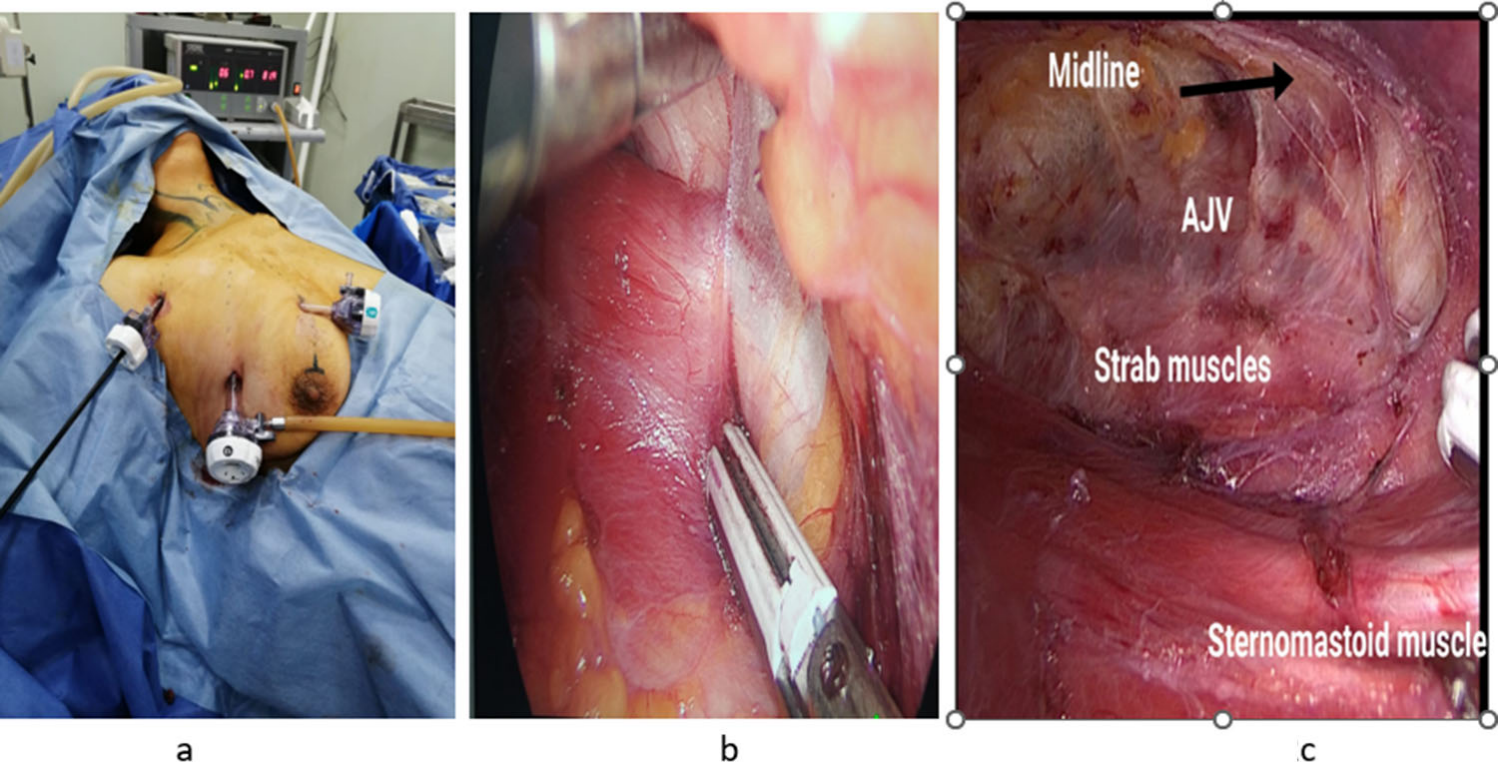
**a** External view of the port sites in unilateral axillo-breast ET approach. **b** Working space creation and identification of sternomastoid tendon. **c** Boundaries of the working space

After disinfection and draping, a 12 mm surgical incision is made at the site of the camera port. This is followed by blunt dissection in the subpectoral fascial plane using a long Kelly clamp to create a preliminary adequate infraclavicular working space. The 12 mm camera port is then introduced followed by a low-pressure CO_2_ insufflation (pressure is set at 8 mmHg, and the flow is set at 1–3 L/min). After that a10 mm 30-degre endoscope is introduced, followed by the other two working ports. For vessel sealing throughout the procedure, we usually use a 5 mm laparoscopic vessel sealer/divider (Ligasure with a blunt or dolphin tip Covidien, M A) (Fig. 2a, b, c).

*Step 3:* Exposure and partial delivery of the thyroid lobe

**Fig. 3:**
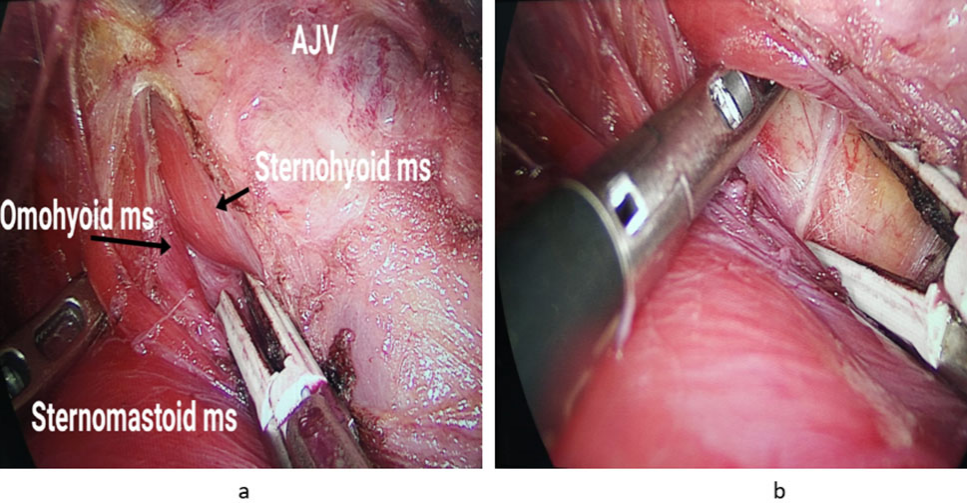
**a** Lateral route of access to the right thyroid lobe in between strab muscles. **b** Right Middle thyroid vein identification

This step is started by dividing the deep fascia between the anterior border of sternomastoid muscle and the adjacent lateral border of the strap muscles. This is followed by splitting the strap muscles over between omohyoid and sternohyoid muscles then across the fibers of sternothyroid muscle (Fig. 3a).

Fine dissection and downward peeling of the false thyroid capsule is then attempted (capsular dissection), to enter the natural holy avascular space between the false and true thyroid capsules, until the middle thyroid vein (MTV) is encountered and subsequently sealed and divided (Fig. 3b).

*Step 4:* Lower thyroid pole dissection with identification and protection of the inferior parathyroid gland

**Fig. 4:**
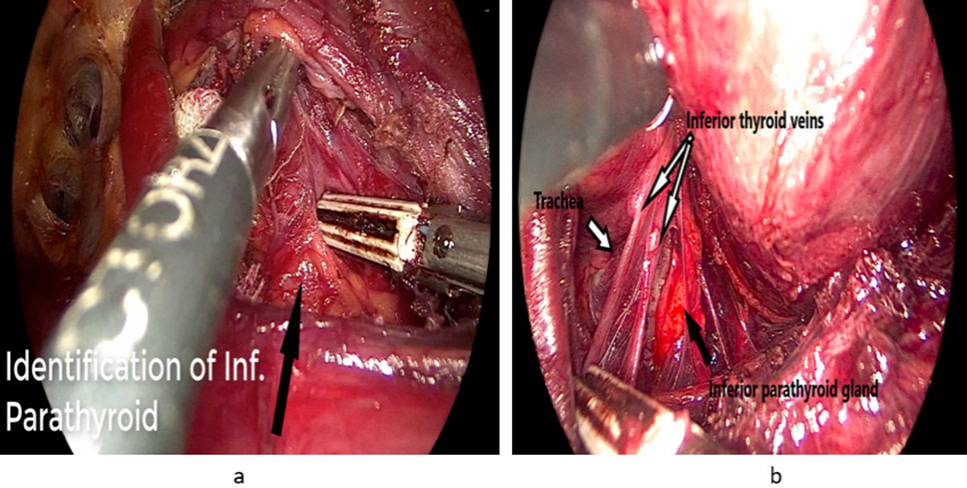
**a** Lower thyroid pole dissection and right inferior parathyroid gland localization (Black arrow). **b** Photo depicts the left inferior parathyroid gland together with inferior thyroid veins and trachea

Combined good and gentle retraction of the partially mobilized lobe is now exerted with one of the working instruments, then dissection of the lower pole of the lobe is continued aiming at identification of the lower pole together with visualization of the inferior thyroid vasculature and inferior parathyroid gland (Fig. 4).

*Step 5:* Proper tracheal exposure

**Fig. 5:**
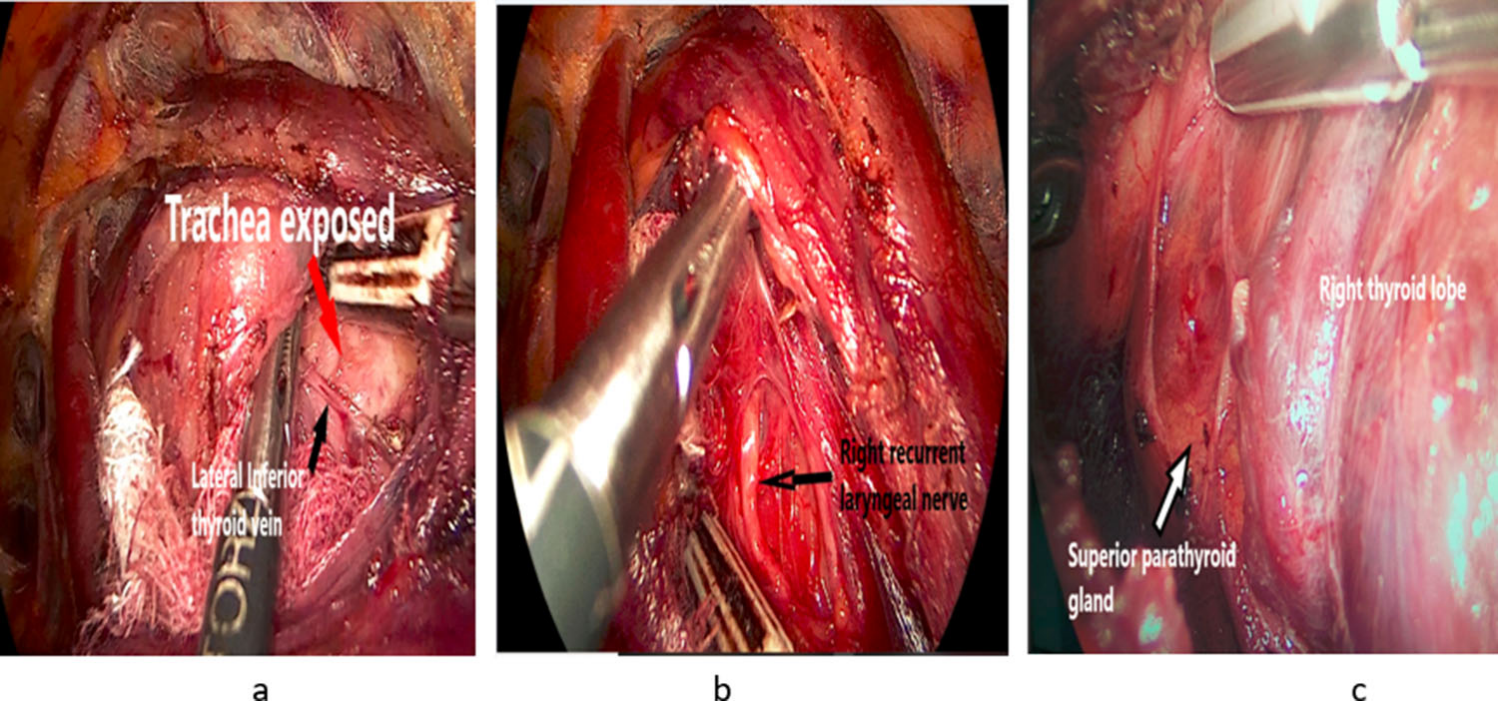
**a** Sealing and dividing of the inferior thyroid veins (black arrow) with subsequent proper tracheal exposure (red arrow). **b** Identification and dissection of right recurrent laryngeal nerve (RLN). c Superior parathyroid localization and preservation

This can be achieved after sealing and dividing the medial and lateral inferior thyroid veins. We consider this step as one of the cornerstone steps in the procedure, as good exposure of the anterior surface of the trachea below the thyroid isthmus is mandatory to predict the possible plane for localization of the recurrent laryngeal nerve (RLN) which usually runs in a plane just below that of the surface of the trachea (Fig. 5a)

*Step 6:* Recurrent laryngeal nerve identification and dissection

After proper tracheal exposure and division of the inferior thyroid veins, gentle medial and upward traction of the target lobe will bring the inferior parathyroid gland in full sight.

Careful gentle downward dissection of the inferior parathyroid gland off the true thyroid capsule is then attempted with preservation of the delicate blood supply to the parathyroid gland. After competition of this step, the inferior thyroid artery glandular branches could be visualized. Gentle dissection in plane below or between these branches will expose the RLN which is then followed cephalad for a short distance till approaching the berry’s ligament (Fig. 5b).

*Step 7:* Identification and protection of the superior parathyroid gland

Dissection is then shifted toward the upper aspect of the target lobe. With good medial traction of the upper part of the target lobe, the superior parathyroid gland with its sentinel fat pad could be identified and preserved (Fig. 5c).

*Step 8:* Proper exposure of the inferior pharyngeal constrictor muscle and upper pole dissection and division

**Fig. 6:**
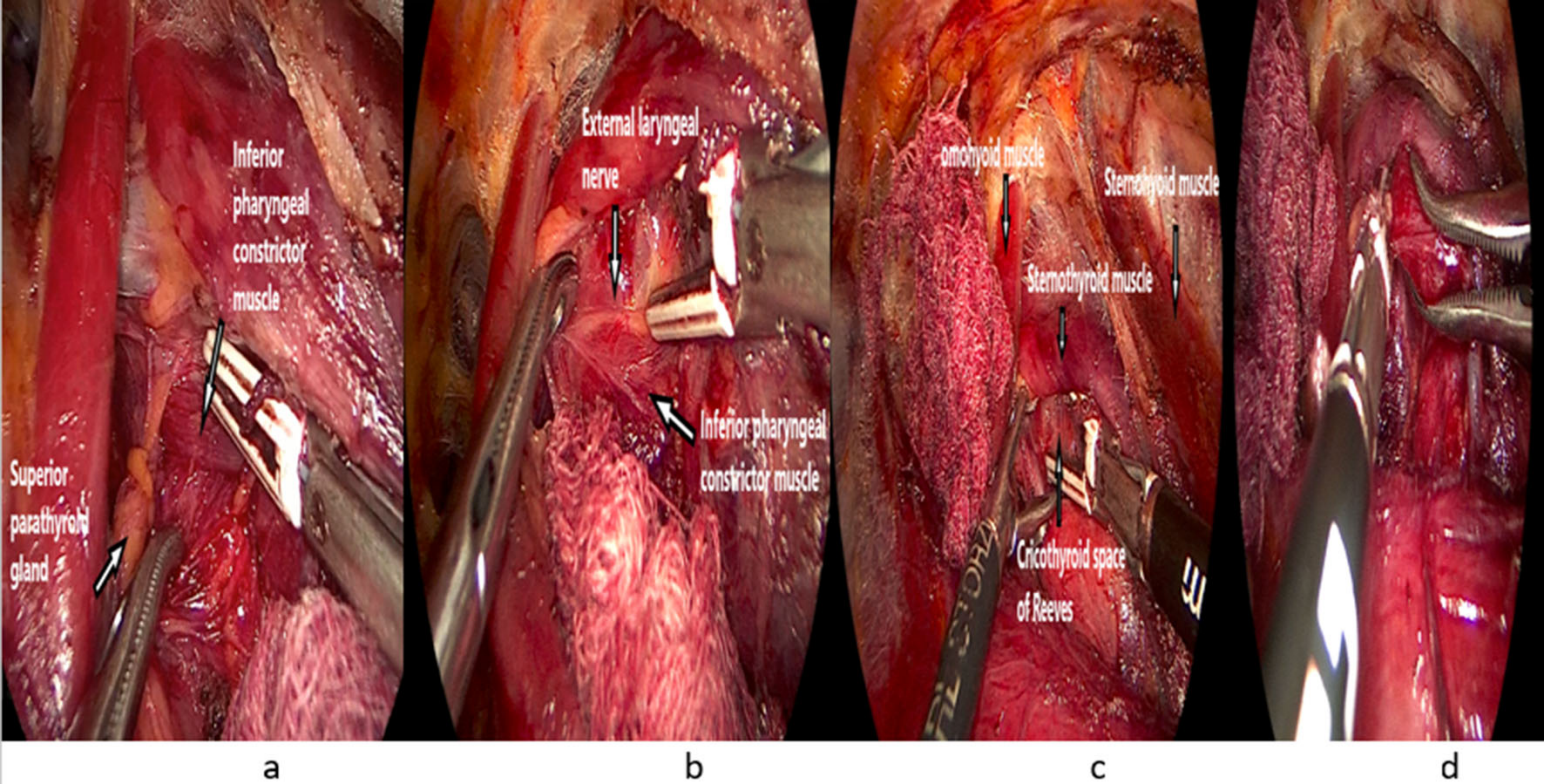
**a** Dissection and proper exposure of the right inferior pharyngeal constrictor. **b** Identification and preservation of right external laryngeal nerve. **c** Dissection of the cricothyroid space of Reeves medial to the upper thyroid lobe. **d** Sealing and dividing the superior thyroid pole vasculature

This is another cornerstone step in the procedure, in which we follow the lower border of the upper thyroid pole by dissecting the loose areolar tissue in this region with sealing of the small, encountered blood vessels until the fibers of inferior pharyngeal constrictor muscle (IPCM) of the pharynx is identified (Fig. 6a).

Further IPCM exposure and gentle dissection (without violation of its fibers), till IPCM-cricothyroid junction, should be carried out aiming at the creation of a good window below the upper pole and its vasculature. This step must be done cautiously and gently to identify and safeguard the external laryngeal nerve (ELN) which often runs over the ICPM below the upper pole vasculature (Fig. 6b).

Fine dissection of the cricothyroid space of Reeves medial to the upper thyroid lobe is then carried followed by careful sealing and division of the upper pole vasculature (Fig. 6c, d).

*Step 9:* Creation of the butterfly critical view of safety (Butterfly CVS)

**Fig. 7:**
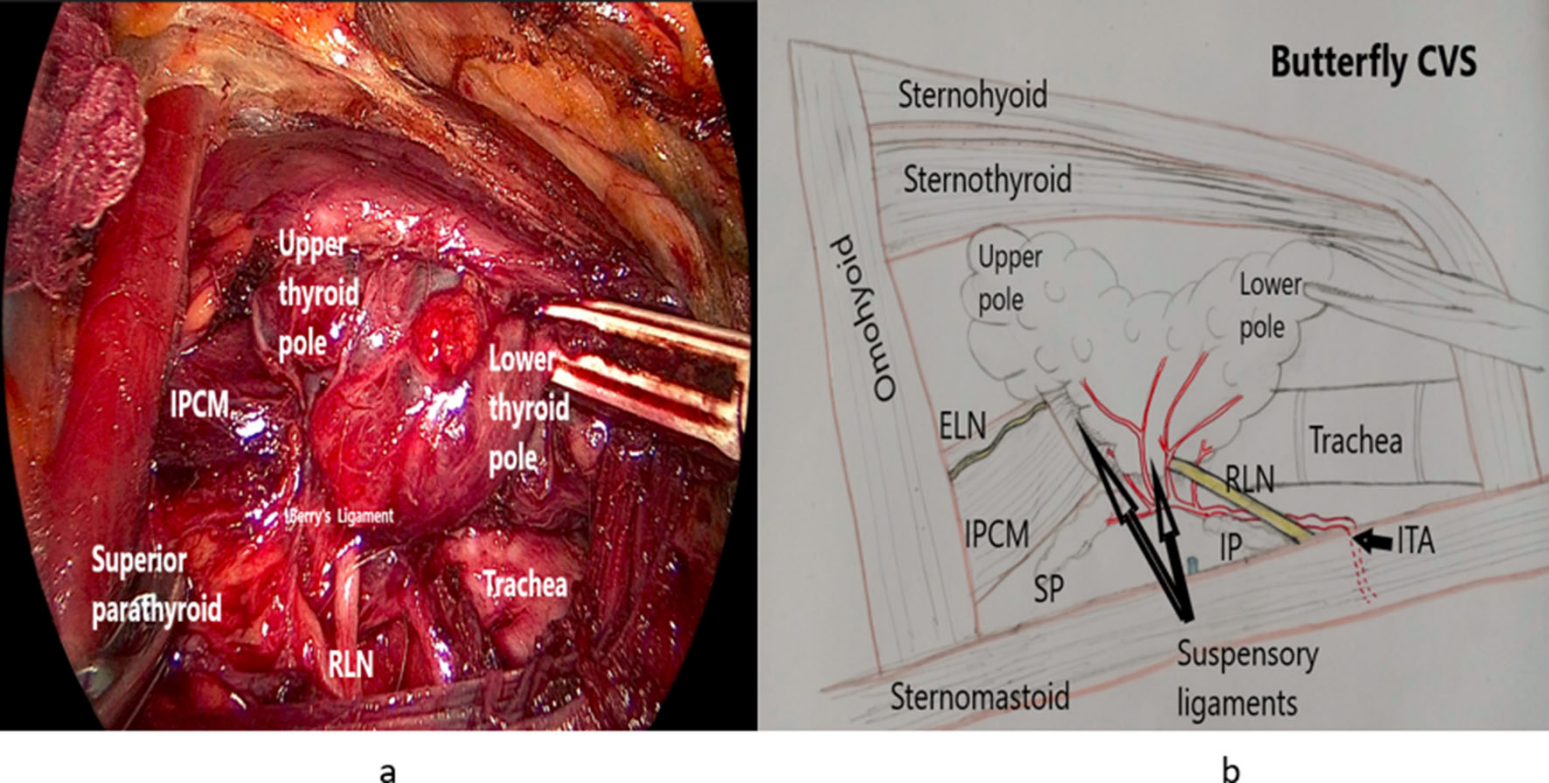
**a** Intraoperative view of the butterfly critical view of safety created before attempting dividing the suspensory ligaments. **b** Diagram illustration of the butterfly critical view of safety (CVS—critical view of safety, RLN—recurrent laryngeal nerve, ELN—external laryngeal nerve, IPCM—inferior pharyngeal constrictor muscle, SP—superior parathyroid gland, IP—inferior parathyroid gland, ITA—inferior thyroid artery)

After complete freeing of the upper and lower thyroid poles, the lobe is now attached only by the suspensory ligaments (the posterior suspensory Berry’s and the superior suspensory ligaments) and the isthmus. By this time, the butterfly CVS can be identified as follows: the upper and lower poles of the target lobe will be floating like the upper wings of a butterfly with berry’s ligament representing the body of the butterfly and lower wings represented by the lower and upper parathyroid glands (the C shaped edge of the thyroid mesentery)[3] with RLN seen running toward the attached berry’s ligament (Fig. 7a, b).

*Step 10:* Further RLN dissection, suspensory ligaments, and isthmus division

**Fig. 8:**
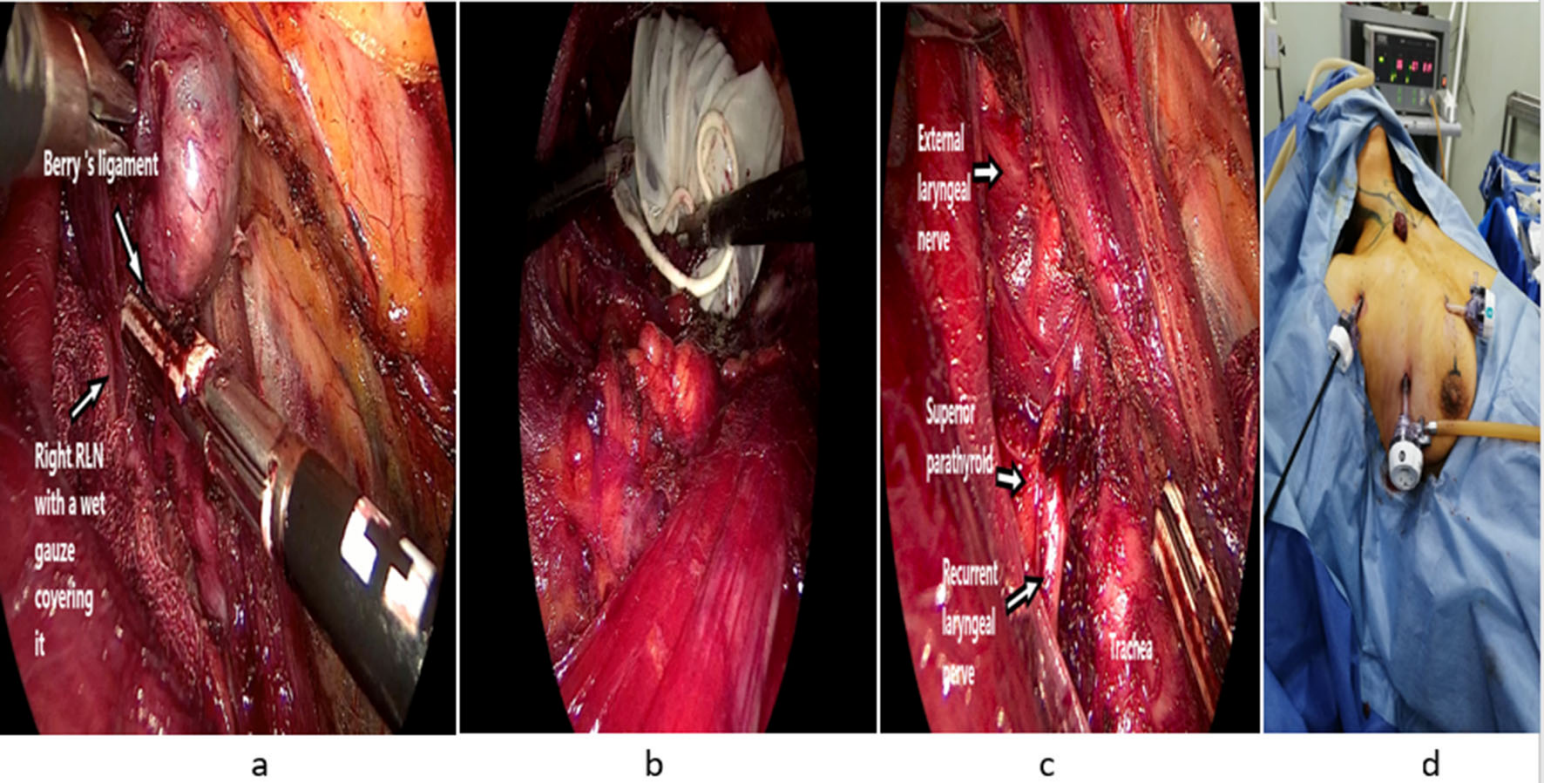
**a** Finalizing RLN dissection and berry’s ligament division. **b** Right thyroid lobe retrieval in a sterile glove. **c** The operative field after right thyroid lobe retrieval. **d** External view after right thyroid lobe retrieval

After proper creation of the butterfly CVS, care is directed toward further cranial dissection of RLN and division of the terminal branches of the inferior thyroid artery which are either passing underneath or above the RLN. Then, layer by layer transection of posterior suspensory Berry’s ligament is performed keeping a small wet gauze over the RLN to protect it from any potential transmitted thermal effect (Fig. 8a). The superior suspensory ligament is then divided followed by isthmus division and target thyroid lobe becomes completely free and ready to be retrieved in a sterile glove or endobag through the camera port (Fig. 8b). Lastly, saline irrigation is done, and meticulous hemostasis is checked, and a suction drain is inserted to be removed after 2–3 days postoperatively (Fig. 8c, d).

## Supplementary Information

Below is the link to the electronic supplementary material.

Supplementary file1 (MP4 149854 KB)

## Data Availability

All the data included are available at our registry (database) system at oncology center Mansoura University.
